# Ultrahigh-Throughput Sample Analysis Using Liquid Atmospheric Pressure Matrix-Assisted Laser Desorption/Ionization Mass Spectrometry

**DOI:** 10.1021/acs.analchem.1c05614

**Published:** 2022-03-02

**Authors:** Henriette Krenkel, Jeffery Brown, Keith Richardson, Emmy Hoyes, Michael Morris, Rainer Cramer

**Affiliations:** † Department of Chemistry, University of Reading, Whiteknights, Reading RG6 6DX, U.K.; ‡ Waters Corporation, Stamford Avenue, Wilmslow SK9 4AX, U.K.

## Abstract

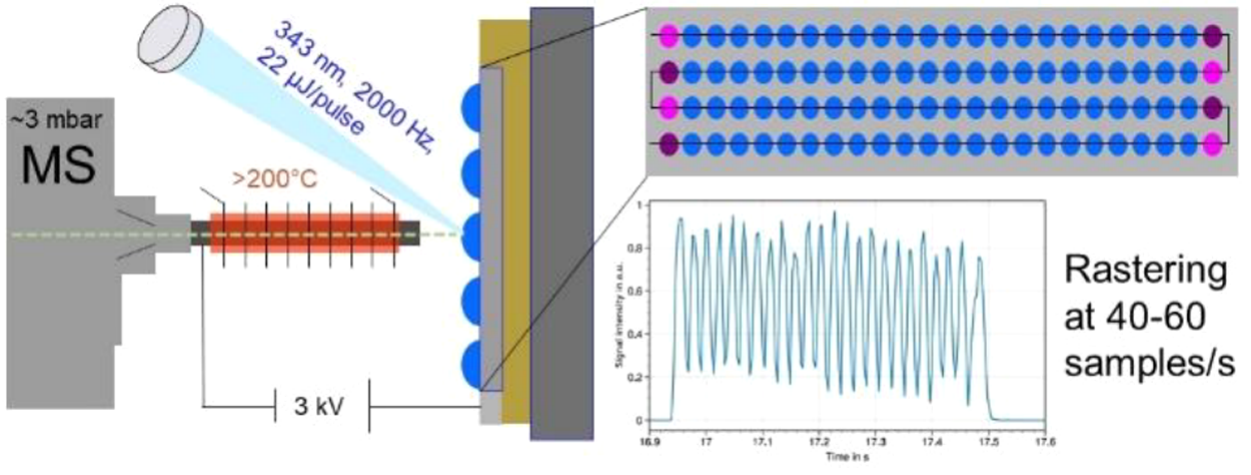

Mass spectrometry (MS) allows for automated analysis of complex samples at high resolution without the need for labeling/derivatization. Liquid atmospheric pressure matrix-assisted laser desorption/ionization (LAP-MALDI) enables rapid sample preparation and MS analysis using microtiter-plate formats and high-performing mass spectrometers. We present a step change in high-speed, large-scale MS sample analysis of peptides at 20 samples/s and an enzymatic assay at 40 samples/s, i.e., an order of magnitude faster than current MS platforms. LAP-MALDI requires only low amounts of sample volume (<2 μL), of which only a fraction (<1%) is typically consumed, and allows for multiplexing and high-speed MS/MS analysis, demonstrated at ∼10 samples/s. Its high ion signal stability and similarity to electrospray ionization enables CVs below 10% and the analysis of multiply charged peptide ions at these extreme speeds. LAP-MALDI MS fulfills the speed requirements for large-scale population diagnostics and compound screening with the potential of analyzing >1 million samples per day.

Label-free, high-throughput MS analysis has recently pushed the limits of sample throughput for compound library screening and inhibitor studies. Especially ambient ionization techniques such as AMI (Acoustic Mist Ionization)^1^ (2–3 samples/s), MAI (Matrix-Assisted Ionization)^2^ (1 sample/s), DESI (Desorption ElectroSpray Ionization)^3^ (2.7 samples/s), ESI (ElectroSpray Ionization)^4^ (0.4 samples/s), and ADE (Acoustic Droplet Ejection)^5^ (0.45 samples/s), as well as conventional MALDI (Matrix-Assisted Laser Desorption/Ionization)^6^ (2.5 samples/s) or hybrid techniques^7^ (0.5–1.3 samples/s for IR-MALDESI (Infrared Matrix-Assisted Laser Desorption Electrospray Ionization)) have produced encouraging results with respect to analytical speed. Although having clear advantages over the routinely used label-based photometric readouts, MS-based fast analysis applications are still not widely employed.

However, ADE,^8^ as well as LAP-MALDI MS,^9^ recently demonstrated new speeds of up to 6 samples/s, reliably producing stable ion signals that are well-separated from each other. The latter of these two approaches is a new addition to the high-speed MS analysis tools with the inherent speed advantage of laser-based techniques. Additional advantages of LAP-MALDI are high ion signal stability,^10,11^ which is crucial for fast sample scanning, and the production of multiply charged analyte ions,^12^ thus allowing the employment of high-performing mass analyzers such as orbitraps and modern Q-TOF instruments. In combination with its low matrix background, LAP-MALDI facilitates the simultaneous detection of low-molecular weight (metabolites, lipids) and high-molecular weight (peptides, proteins) analytes,^13^ outperforming conventional solid MALDI on axial TOF instruments. LAP-MALDI MS and its associated (offline) upfront sample preparation support large-scale analyses, by using microtiter-plate format and multiple robotic preparation platforms to feed one LAP-MALDI mass spectrometer.

Importantly, LAP-MALDI is inherently fast. On a commercial Q-TOF instrument, recorded ion packets from individual desorption events are <5 ms wide,^14^ allowing an acquisition rate of up to 200 desorption events per second. Thus, we further developed LAP-MALDI with Q-TOF instrumentation by optimizing instrumental bottlenecks such as spectral scan rates, laser repetition rate, sample plate movement, and sample number per plate (see additional experimental details in the Supporting Information), in order to push the speed limits toward tens and ultimately hundreds of samples per second.

## Experimental Section

### LAP-MALDI and MS Setup

The general LAP-MALDI setup can be found elsewhere.^9^ For this work, a diode-pumped solid-state (DPSS) laser was used with a wavelength of 343 nm and a pulse repetition rate of 2000 Hz (Flare NX 343-0.2-2, Coherent, Santa Clara, USA). MALDI sample plates were rastered as described in the Supporting Information. The acquisition mode SONAR^15^ (Waters) was used with the quadrupole scanning being disabled and in RF-only mode. Ion mobility gases were turned off. Each of the SONAR TOF ‘scans’ were stored in 200 consecutive spectra or ‘bins’, allowing the acquisition and storage of up to 1000 spectra/s, while the temporal resolution increased up to 0.93 ms per spectrum/bin.

### Matrix Preparation and Sample Spotting

CHCA was dissolved in acetonitrile and water (1:1; v/v) to a concentration of 5 mg/mL. After short sonication, propylene glycol (PG) was added at 60% by volume. The matrix was mixed with a sample at a ratio of 1:1 (v/v), and 1 or 0.3 μL of the mixture was spotted onto the stainless-steel sample plate using a 384- or 1536-well format, respectively.

### Peptide Analysis

A total of 10 pmol of peptide was used for each LAP-MALDI sample. The MALDI samples were analyzed in each row by moving the sample plate at a constant speed of 50–200 mm/s. To ease postacquisition data processing, the start and end of each sample row was marked with a sample using the analyte standard Angiotensin I (Ang I) (40 pmol) and *N*-Hippuryl-His-Leu hydrate (HHL) (10 pmol), respectively.

### Enzyme Assay

Angiotensin-converting enzyme (ACE) was dissolved in 50 mM Tris buffer at pH 8.5 to yield 0.1 U/mL and mixed with the substrate 1:1 (v/v, 320 pmol/μL Ang I or 100 pmol/μL HHL). The mixture was incubated at 37.5 °C for several hours.

Additional experimental details can be found in the Supporting Information.

## Results and Discussion

For initial testing, a 384-well microtiter-format sample plate was prepared by alternatingly spotting two peptides: bradykinin (Brdk) and [Lys-des-Arg^9^]-bradykinin (Lys-Brdk). The sample plate was automatically rastered with an analysis speed of >20 samples/s per row. The overall speed for the sample plate was slightly reduced to 17 samples/s, as additional time was needed to move from the last sample in each row to the first sample in the next row due to restrictions in the sample stage movement and data processing software (see additional experimental details in Supporting Information). The total data acquisition time for an entire sample plate based on the microtiter-plate format was less than 23 s. Figure S1 shows a diagrammatic scheme of the LAP-MALDI source as used in this study.

**Figure 1:**
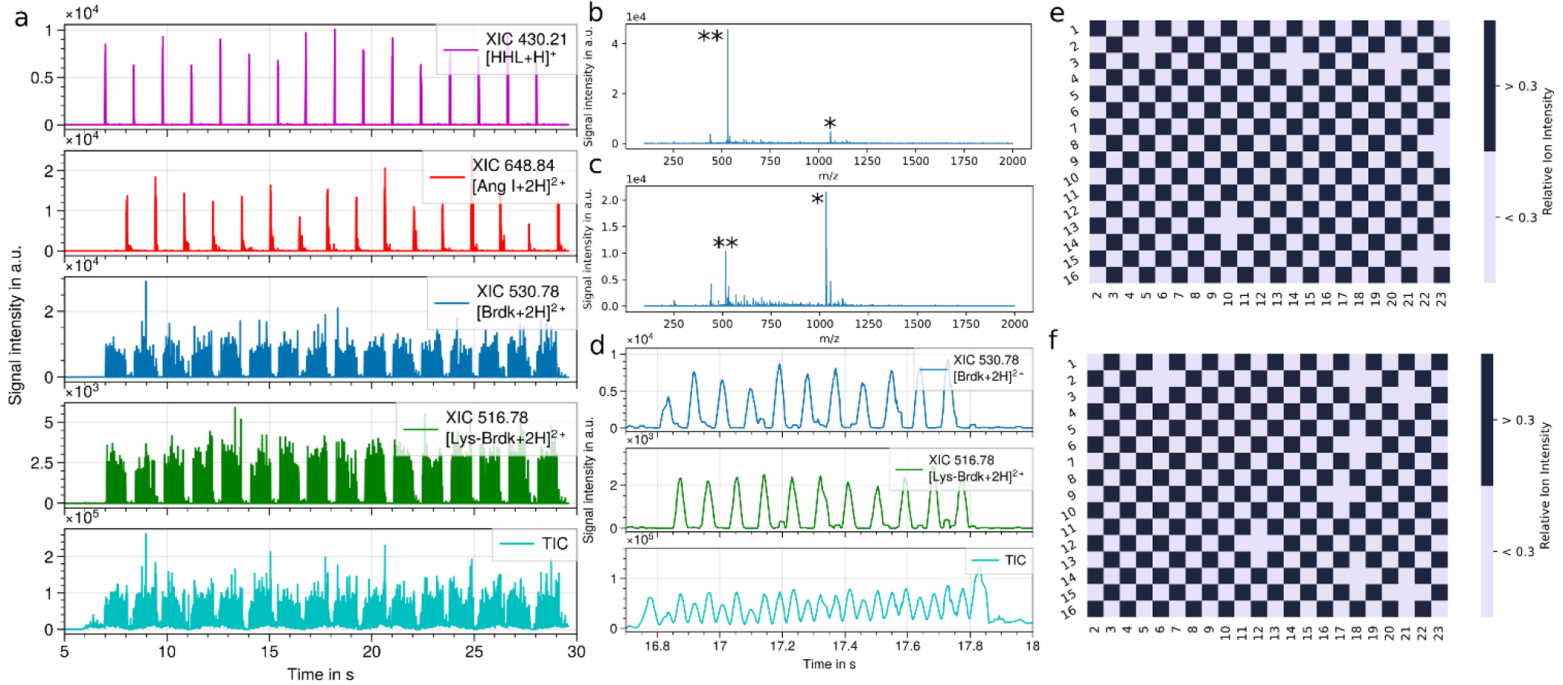
LAP-MALDI MS analysis of a 384-well plate with alternating Brdk and Lys-Brdk samples and different standards at the start and end of each sample row at 20 samples/s for each row (average of 17 samples/s for the entire plate). a) From top to bottom: extracted ion chromatogram (XIC) of [HHL+H]^+^ (*m*/*z* 430.21, start marker), [Ang+2H]^2+^ (*m*/*z* 648.84, end marker), [Brdk+2H]^2+^ (*m*/*z* 530.78), [Lys-Brdk+2H]^2+^ (*m*/*z* 516.78), and total ion chromatogram (TIC). Mass spectra of first (b) (Brdk) and last (c) (Lys-Brdk) peptide sample of the plate. Singly (*) and doubly (**) charged analyte ions are labeled. d) Enlargement of one row of analyte ion signals. From top to bottom: XIC of [Brdk+2H]^2+^ (*m*/*z* 530.78), [Lys-Brdk+2H]^2+^ (*m*/*z* 516.78), and total ion chromatogram (TIC). Analyte ion signal intensities for Brdk (e) and Lys-Brdk (f) using a 30% threshold.

The data obtained from this initial analysis clearly show well-separated ion signals for all samples without any analyte carryover (see Figure 1a–d). As previously reported, predominantly doubly charged peptide ions are observed.^12^ Mass spectrometer scan rates were adjusted to yield at least 10 data acquisitions across each sample, keeping the detection deadtime (interscan delays; ISDs) to a minimum. This is important as data acquisition gaps caused by ISDs lead to the loss of some or all ion signals from a sample.

Heatmaps for both peptides using simple ion signal intensity thresholds (see Figure 1e,f) reveal a detection (classification) accuracy of >95% over multiple analyses (*n* = 3, see Figure S2). Greater accuracy is achieved at slightly lower throughput (∼99% at 10 samples/s, *n* = 3, see Figure S3). Sample position effects, e.g., edge effects, are not evident (see Figure S4). The few failures in the correct sample assignment are currently mostly due to sample spotting, which is envisaged to be improved by adequate robotic liquid handlers capable of handling small volumes of liquid (<1 μL) for MALDI sample preparation. To further automate the entire workflow, plate-changing robotics can be used, which can achieve sample plate-swapping in around 5 s.^16^ Overall high-throughput analysis time could therefore be around 30 s per plate, allowing 120 plates to be analyzed per hour with an adequate multistage robotic feeding system. In 24 h, more than a million samples could be screened, in principle.

**Figure 2:**
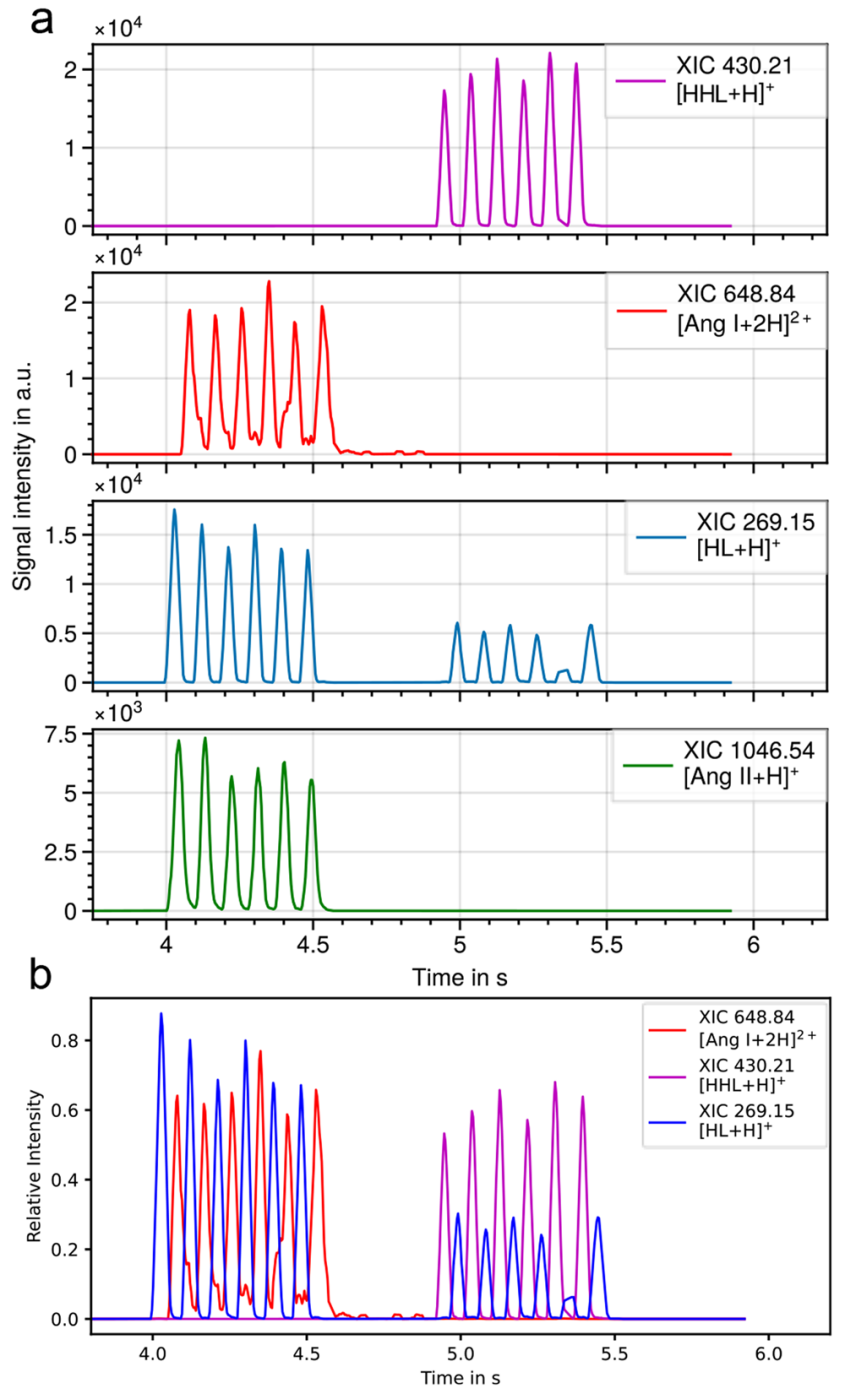
LAP-MALDI MS analysis of an angiotensin-converting enzyme assay with alternating enzyme-treated and untreated samples for Angiotensin I (Ang I, first row) and *N*-Hippuryl-His-Leu hydrate (HHL, second row). a) From top to bottom: XIC of [HHL+H]^+^ (*m*/*z* 430.21, substrate), [Ang I + 2H]^2+^ (*m*/*z* 648.84, substrate), His-Leu (*m*/*z* 269.15, product), and Angiotensin II [Ang II + H]^+^ (*m*/*z* 1046.54, product). b) Overlay of chromatograms shown in a. Average data acquisition speed was 16 samples/s using a stage speed of 100 mm/s.

This level of sample throughput is desirable for compound and assay screening in pharmaceutics. Consequently, the platform’s applicability to enzyme assays was also tested by monitoring the conversion of an enzyme substrate and its product’s appearance simultaneously (see Figure 2). ACE showed full transformation of its natural substrate Ang I and HHL, commonly used in fluorescence experiments. The substrate ion signal intensity substantially decreased after enzyme treatment, and new ion signals at *m*/*z* 269.1589 and *m*/*z* 1046.5422 appeared, which can be attributed to cleaved protonated His-Leu and converted Angiotensin II (Ang II) with a mass measurement accuracy of 7 and 4 ppm, respectively. Although some ion suppression due to the buffer can be observed (data not shown), reproducible and visibly time-resolved peaks were observed at the same acquisition speed as used for the peptide standards (see Figure 2). With increasing mass (mainly above 1000 Da), we observe the appearance of additional (temporally delayed) low-intensity analyte ion peaks at these high-speed acquisitions. These are most likely due to the complex ion path within the employed Q-TOF/ion mobility instrument and are currently under investigation.

To further increase sample throughput, two other bottlenecks were addressed. First, the speed of the translational stage for sample plate movement was increased by a factor of up to 4 (from 50 to 200 mm/s). Second, the sample plate layout was changed to the 1536-well format. Samples were spotted closer to each other and made smaller as actual sample consumption per desorption event is minimal (less than 1‰^12^).

**Figure 3:**
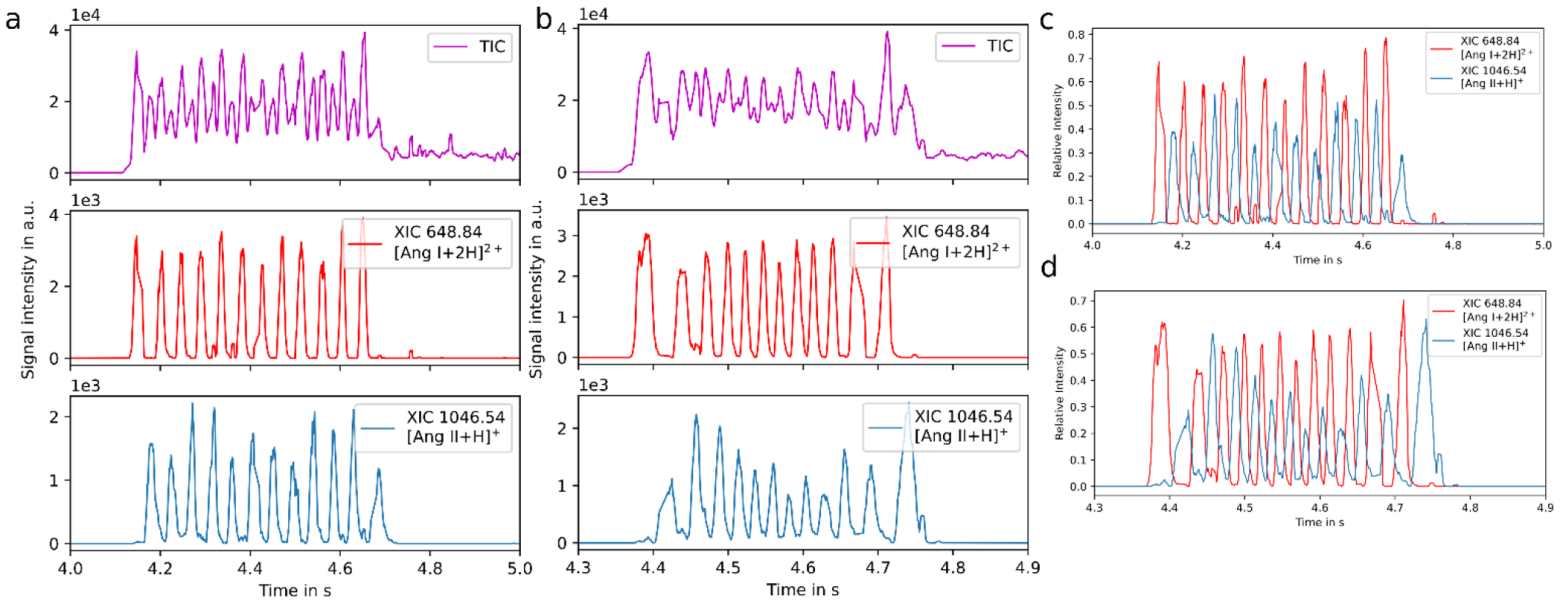
LAP-MALDI MS analysis of an angiotensin-converting enzyme assay alternating enzyme-treated and untreated samples for Angiotensin I using a 1536-well plate layout. a) 40 samples/s (stage movement speed of 100 mm/s); b) 60 samples/s (stage movement speed of 200 mm/s); c) overlay of XICs shown in a; d) overlay of XICs shown in b. TIC: total ion chromatogram; XIC: extracted ion chromatogram.

Tighter sample spotting and a stage speed of 200 mm/s were first tested with the ACE assay (see Figure 3). These changes led to well-resolved peaks at >40 samples/s. With faster sample movement (≥200 mm/s), acceleration and deceleration of the translational stage clearly show a broadening effect for the first and last samples in each row. In addition, the above-mentioned double-peaking for higher masses and the reduced number of sampling points per sample currently limit the maximum analysis speed to 40 samples/s for analytes with a mass >1000 Da. Interestingly, the analysis of the lower-mass substrates (*m*/*z* 430.21) and products (*m*/*z* 269.15) clearly shows baseline-resolved analyte ion signals between the alternating samples at a speed of 40 samples/s (Figure 3a).

**Figure 4:**
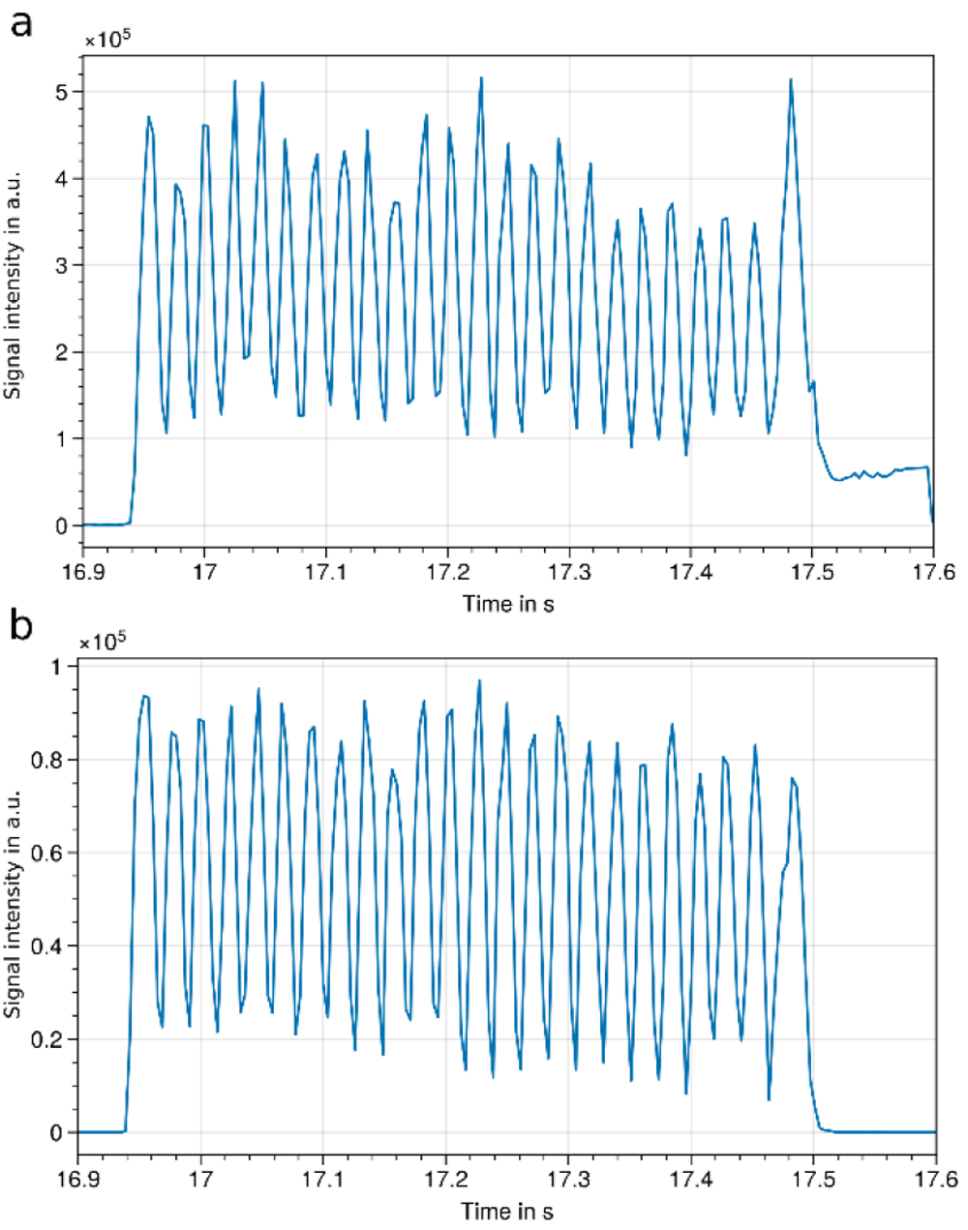
LAP-MALDI MS analysis of HHL using a 1536-well plate layout. a) TIC and b) XIC of [HHL+H]^+^ at 41 samples/s. TIC: total ion chromatogram; XIC: extracted ion chromatogram.

Next, extreme sample throughput levels were tested by analyzing HHL (see Figure 4). While the first and last samples in each row are only slightly broadened due to stage acceleration/deceleration at a speed around 40 samples/s, peak broadening worsens and expands to other samples at even higher speeds. Nonetheless, samples can still be separated in their TICs (total ion chromatograms) and XICs (extracted ion chromatograms) at 60 samples/s (see Figure S5a,b). By analyzing several samples of Brdk multiple times, coefficients of variation (CVs) below 10% (<5% for [HHL+H]^+^) were achieved at conditions corresponding to a speed of 9 samples/s (see Figure S6). Other MS-based techniques result in similar variability but at significantly lower sample throughput.^17,18^ Currently, higher speeds result in higher CVs, e.g., 15% at 20 samples/s.

Finally, ultrahigh-throughput LAP-MALDI tandem mass spectrometry (MS/MS) was demonstrated by analyzing Brdk and its collision-induced dissociation (CID) fragment ions using a fixed window quadrupole. Typical CID peptide fragment ions such as y- and b-type ions were detected at a speed of 12 samples/s per row (see Figure S7). This initial data shows the potential of LAP-MALDI for high-speed SRM/MRM applications.

## Conclusion

In summary, we have shown a step change in the speed for analyzing individual samples by mass spectrometry. So far, up to 60 samples per second can be well separated at the fwhm level by their analyte ion signal. These significant speed increases by an order of magnitude or more compared to previously published reports were achieved by overcoming several bottlenecks such as data acquisition speed (adjusted for ISD deadtimes), laser pulse repetition rate (increased to 2,000 Hz), and sample plate stage speed (increased to 200 mm/s) as well as tighter sample spotting and the use of liquid matrices with their high MALDI ion signal stability. Without the latter, the practical analysis speed would be severely compromised due to the fluctuating ion signal, typically observed with MALDI, thus impeding automated data processing. As used in LAP-MALDI MS, liquid matrices also allow for the generation of multiply charged ions, and together with an atmospheric ion source, high-performing mass spectrometers and superior MS/MS analyses can be employed; in addition, sample plates can be exchanged significantly more quickly in AP-MALDI than in vacuum MALDI sources. MS/MS analysis at these extreme speeds will take screening of large sample sets to an even higher level, in particular with respect to specificity and multiplexing using SRM/MRM approaches.

The method described here can be applied to a vast set of analytes, and the demonstrated analysis speed is even greater than the speed reported for MALDI imaging on commercial instrumentation,^19^ which currently achieves around 20 pixels/s (without gaps between samples). Future modifications of the mass spectrometry acquisition software should allow data acquisition without data loss between scans (due to ISD). Lower scan times and thus higher temporal resolution will provide an additional boost to speed. In general, the theoretical speed limit of the method is determined by the temporal width of the ion plume formed during desorption. Further advancements can be made by using adequate robotics for tighter sample positioning (e.g., 6144-well microtiter-plate format) and employing sample stages with even higher speed as well as acceleration/deceleration. In contrast to other techniques like ADE,^18^ sample volumes can be <1 μL, and tighter sample layouts do not result in increased analysis times but lead to a significant throughput improvement.

Potential application areas for LAP-MALDI MS are in large-scale (multiplex) population diagnostics and in screening of compound libraries within the pharmaceutical industry, where a throughput of 1 million samples per day or more is highly desirable.

## Supporting Information Available

Additional experimental details, triplicate measurements of 384-well plates at 2 analysis speeds, and data on variability including edge effects (PDF)

## Notes

Data supporting the results reported in this paper are openly available from the University of Reading Research Data Archive at 10.17864/1947.000338.
