# Supplementary material for: Ultrahigh-Throughput Sample Analysis Using Liquid Atmospheric Pressure Matrix-Assisted Laser Desorption/Ionization Mass Spectrometry
Source: Anal Chem. 2022 Mar 2;94(10):4141–5. doi: 10.1021/acs.analchem.1c05614 (PMC9385107; doi:10.1021/acs.analchem.1c05614)
Supplement: Supplementary file 1 — ac1c05614_si_001.pdf [file ac1c05614_si_001.pdf]

Supporting Information

Ultrahigh-throughput sample analysis using liquid atmospheric pressure (LAP) matrix-assisted laser desorption/ionisation (MALDI) mass spectrometry

*Henriette Krenkel,[1] Jeffery Brown[2], Keith Richardson[2], Emmy Hoyes[2], Michael Morris[2], Rainer Cramer[1]**

[1] Department of Chemistry, University of Reading, Whiteknights, Reading RG6 6DX, UK

[2] Waters Corporation, Stamford Avenue, Wilmslow SK9 4AX, UK

* R. Cramer
Department of Chemistry
University of Reading
Whiteknights, Reading RG6 6DX, United Kingdom
E-mail: r.k.cramer@reading.ac.uk
Homepage: www.reading.ac.uk/chemistry/about/staff/r-k-cramer.aspx

Table of Content

Additional Experimental details **Page S3**

Figure S1: General LAP-MALDI setup: 1) UV laser, 2) liquid sample at atmospheric pressure, 3) sample plate, 4) sample plate holder, 5) 2-dimensional translational stage, 6) inlet tube heated by resistance wire and 7) Q-TOF mass spectrometer. **Page S5**

Figure S2: LAP-MALDI MS analysis of a 384-well plate with alternating Brdk and Lys-Brdk samples and different standards at the start and end of each sample row at 20 samples/s for each row (average of 17 sample/s for the entire plate). Analyte ion signal intensities for Brdk (top) and Lys-Brdk (bottom) for three runs using a 30 % threshold. **Page S6**

Figure S3: LAP-MALDI MS analysis of a 384-well plate with alternating Brdk and Lys-Brdk samples and different standards at the start and end of each sample row at 10 samples/s for each row (average of 9 sample/s for the entire plate). Analyte ion signal intensities for Brdk (top) and Lys-Brdk (bottom) for three runs using a 25 % threshold. **Page S6**

Figure S4: Graphical analysis of analytical performance and variability. LAP-MALDI MS analysis of a 384-well plate with alternating Brdk and Lys-Brdk samples and different standards at the start and end of each sample row at 20 samples/s for each row (average of 17 sample/s for the entire plate). Mean and standard deviation over each column (a,b) and each row (c,d) for Brdk and Lys-Brdk. Heatmaps for absolute ion intensity for Brdk (e) and Lys-Brdk (f). **Page S7**

Figure S5: LAP-MALDI MS analysis of HHL using a 1536-well plate layout. a) total ion chromatogram, and b) extracted ion chromatogram at 60 samples/s. **Page S8**

Figure S6: Variability of peptide analysis in TDC mode. 48 consecutive LAP-MALDI analyses of 2x2 sample spots of Brdk and different standards at start and end at 50 mm/s stage speed. Enlargement on right side. **Page S9**

Figure S7: Tandem mass spectrometry at 50 mm/s. 12 samples of Brdk with quadrupole filtering around m/z 530.78 and trap collisional voltage at 25 V. a) Extracted ion chromatogram of two fragment ions; b) Mass spectrum of first sample; c) Mass spectrum of last sample. **Page S9**

Additional Experimental details

Materials. Angiotensin I (Ang I) was bought from Enzo Life Sciences (Farmingdale, USA). α-Cyano-4-hydroxycinnamic acid (CHCA), propylene glycol (PG), Angiotensin Converting Enzyme from rabbit lung (ACE), N-Hippuryl-His-Leu hydrate (HHL), Bradykinin (Brdk) and [Lys-des-Arg^9]-Bradykinin (Lys-Brdk) were bought from Merck (Darmstadt, Germany). Tris Plus One was purchased from Amersham Biosciences. HPLC-grade water was purchased from Fisher Scientific (Loughborough, UK). Acetonitrile (Chromasolve, HPLC grade) was bought from Honeywell Riedel-de-Haën (Charlotte, USA).

LAP-MALDI and MS setup. The general LAP-MALDI setup can be found elsewhere[1]. Briefly, a Synapt G2-Si (Waters, Wilmslow, UK) was modified with a home-built LAP-MALDI ion source using a heated ion transfer tube. The control and acquisition software used was MassLynx 4.2 (Waters).

Two hardware improvements were necessary to allow higher sample throughput. Firstly, instead of a 30-Hz nitrogen laser, a diode-pumped solid-state (DPSS) laser at 343 nm with a pulse repetition rate of 2000 Hz (Flare NX 343-0.2-2, Coherent, Santa Clara, USA) was used to increase the number of laser shots per sample while decreasing the residence time on each sample spot. Secondly, the target plate speed was increased by using a faster linear actuator (X-LSQ, Zaber, Vancouver, Canada), featuring an encoder for precise position readback.

In general, MALDI sample plates were continuously rastered row-wise by starting the next row with the sample closest to the sample analysed last in the previous row. To achieve the same data acquisition time for each sample, and thus facilitating post-acquisition data processing, the sample raster was adapted to move slightly beyond the end of each row before moving to the next, thus avoiding prolonged residence time on the last sample due to the deceleration needed for turning to the next row. As additional time was therefore needed to move from the last sample in each row to the first sample in the next row, there was a slightly reduced overall speed for the analysis of an entire plate.

A 384-well MALDI target plate in the microtiter plate-format was used to allow easy interfacing with standard robotics and sample preparation. Therefore, a 3d-printed holder was designed to allow the use of standard and non-standard target plates.

Due to data acquisition rate limitations, the commercial instrument software does not provide a readout for every single ToF spectrum (as the oaTOF is pushing ion packets at tens of kHz). Instead, an appropriate accumulation of ToF spectra is obtained for each spectral "scan". For the Synapt, the MassLynx 4.2 ToF spectral accumulation time has a minimum setting of 16 ms per scan and an InterScan Delay (ISD) of at least 10 ms between every scan. This does not allow sufficient temporal resolution for ultrafast throughput analysis. Hence, the recently developed acquisition mode SONAR[2] (Waters) was used in an adapted way. The quadrupole scanning was disabled and used in RF-only mode and ion mobility gases were turned off, while TRAP TWAVES were optimised to guide ion packages through at maximum speed. Each of the SONAR ToF "scans" were stored in 200 consecutive spectra or "bins". Thus, ISDs occur after each scan but not after each spectrum/bin. As a consequence, if the scan time was set at 0.2 seconds (for example), individual consecutive spectra could be acquired and stored at approximately 1000 spectra/s and the temporal resolution increased to 0.93 ms per spectrum/bin.

Data processing. Raw files recorded in the adapted SONAR format were converted to MassLynx-readable files using a custom-made software (for software access contact K.R.). Ion signals for each sample were automatically detected and spatially labelled by custom-made data processing software (for software access contact E.H.) by detecting marker ion signals at the start and end samples for each row and evenly distributing the number of scans between into the specified number of samples with the help of MassLynx SDK. Separate files were created for each sample's ion signals, allowing further standard data post-processing. Data were smoothed using Savitzky-Golay algorithm. Ion intensities for heatmaps were extracted using specproc (https://sourceforge.net/projects/specproc/).

Matrix preparation and sample spotting. CHCA was dissolved in acetonitrile and water (1:1; v/v) to a concentration of 5 mg/mL. After short sonication, 60 % PG was added by volume. The matrix was mixed with sample at a ratio of 1:1 (v/v), and 1 µL or 0.3 µL of the mixture was spotted onto the stainless-steel target plate using a 384- or 1536-well format, respectively.

Peptide analysis. A total of 10 pmol of peptide was used for each LAP-MALDI sample. The MALDI samples were analysed in each row by moving the sample plate at a constant speed of 50-200 mm/s. To ease post-acquisition data processing, the start and end of each sample row was marked with a sample using the analyte standard Ang I (40 pmol) and HHL (10 pmol), respectively.

Enzyme assay. ACE was dissolved in 50-mM tris buffer at pH 8.5 to yield 0.1 U/mL and mixed with substrate 1:1 (v/v, 320 pmol/µL Ang I or 100 pmol/µL HHL). The mixture was incubated at 37.5°C for several hours.

Coefficient of Variation (CV) measurements. Four samples of Brdk were spotted on the target plate in rows of two. HHL and Ang I were used for marking each row as described above. The translational stage was set to cycle trough the samples to measure Brdk 96 times. The detector mode was set to time-to-digital-converter (TDC). Peak detection was performed in MassLynx (Savitzky-Golay smooth, peak separation 30 %, peak threshold 10 % relative height).

Tandem mass spectrometry. Twelve samples of Brdk (20 pmol each on target) were analysed with a fixed quadrupole window around m/z 530.78. A collisional voltage in the trap cell of 25 V was applied.

References

1. Krenkel, H.; Hartmane, E.; Piras, C.; Brown, J.; Morris, M.; Cramer, R., Advancing Liquid Atmospheric Pressure Matrix-Assisted Laser Desorption/Ionization Mass Spectrometry Toward Ultrahigh-Throughput Analysis. *Analytical Chemistry* **2020,** *92* (4), 2931-2936.
2. Gethings, L. A.; Richardson, K.; Wildgoose, J.; Lennon, S.; Jarvis, S.; Bevan, C. L.; Vissers, J. P. C.; Langridge, J. I., Lipid profiling of complex biological mixtures by liquid chromatography/mass spectrometry using a novel scanning quadrupole data-independent acquisition strategy. *Rapid Communications in Mass Spectrometry* **2017,** *31* (19), 1599-1606.

Figures

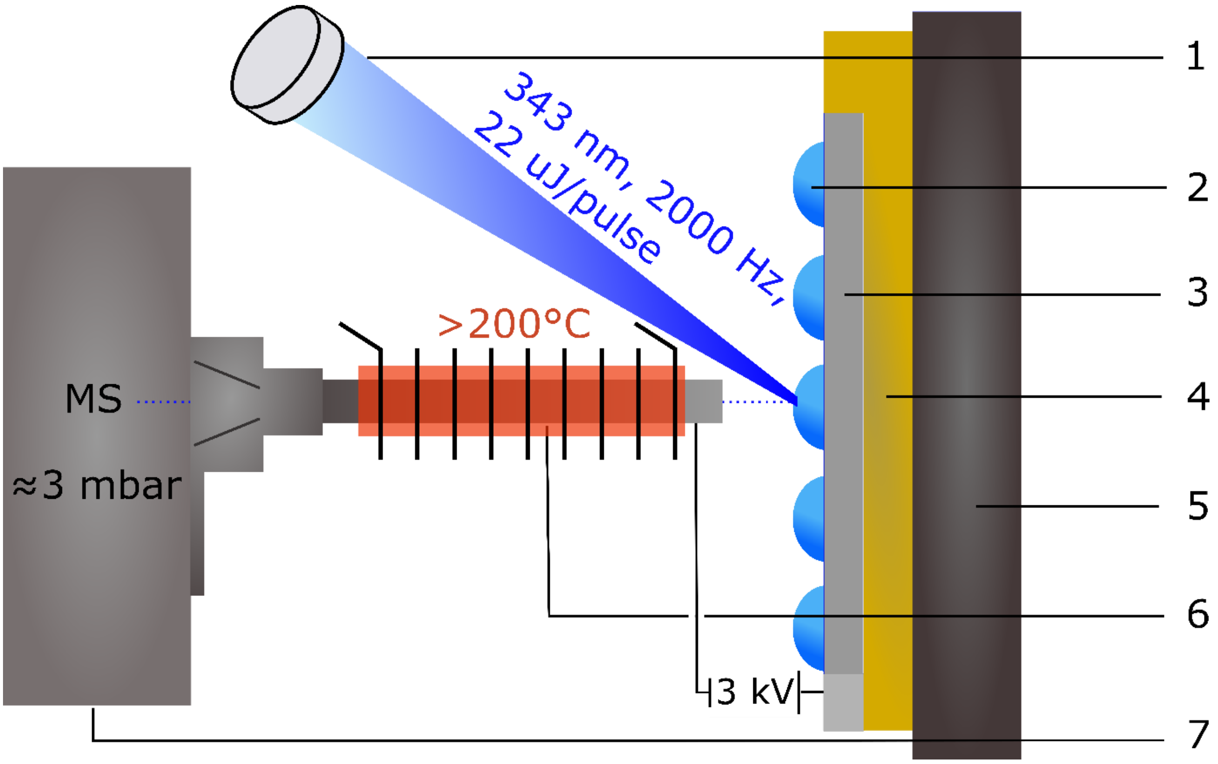

Figure S1: General LAP-MALDI setup: 1) UV laser, 2) liquid sample at atmospheric pressure, 3) sample plate, 4) sample plate holder, 5) 2-dimensional translational stage, 6) inlet tube heated by resistance wire and 7) Q-TOF mass spectrometer

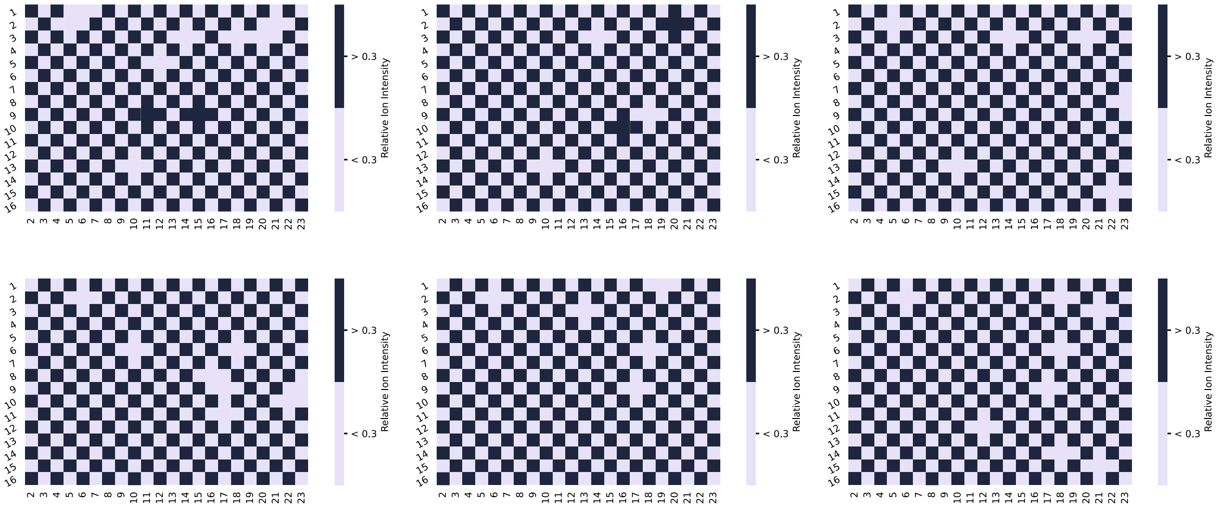

Figure S2: LAP-MALDI MS analysis of a 384-well plate with alternating Brdk and Lys-Brdk samples and different standards at the start and end of each sample row at 20 samples/s for each row (average of 17 sample/s for the entire plate). Analyte ion signal intensities for Brdk (top) and Lys-Brdk (bottom) for three runs using a 30 % threshold.

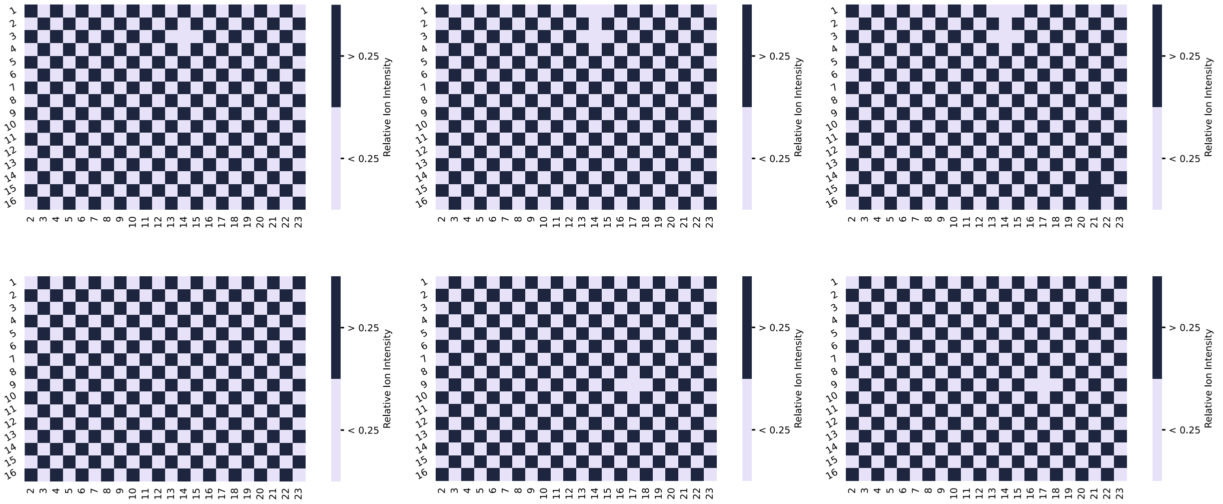

Figure S3: LAP-MALDI MS analysis of a 384-well plate with alternating Brdk and Lys-Brdk samples and different standards at the start and end of each sample row at 10 samples/s for each row (average of 9 sample/s for the entire plate). Analyte ion signal intensities for Brdk (top) and Lys-Brdk (bottom) for three runs using a 25 % threshold.

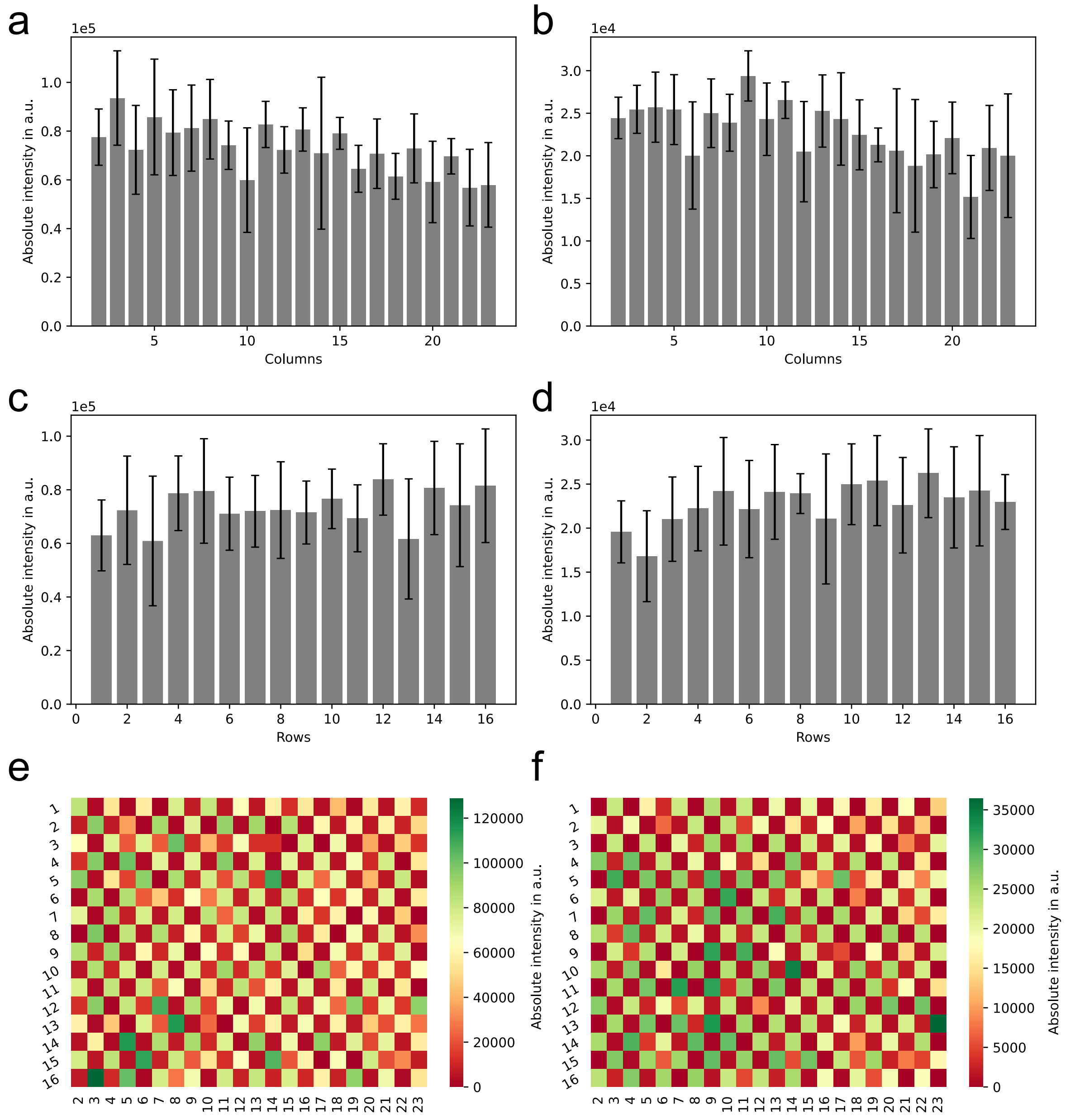

Figure S4: Graphical analysis of analytical performance and variability. LAP-MALDI MS analysis of a 384-well plate with alternating Brdk and Lys-Brdk samples and different standards at the start and end of each sample row at 20 samples/s for each row (average of 17 sample/s for the entire plate). Mean and standard deviation over each column (a,b) and each row (c,d) for Brdk and Lys-Brdk. Heatmaps for absolute ion intensity for Brdk (e) and Lys-Brdk (f).

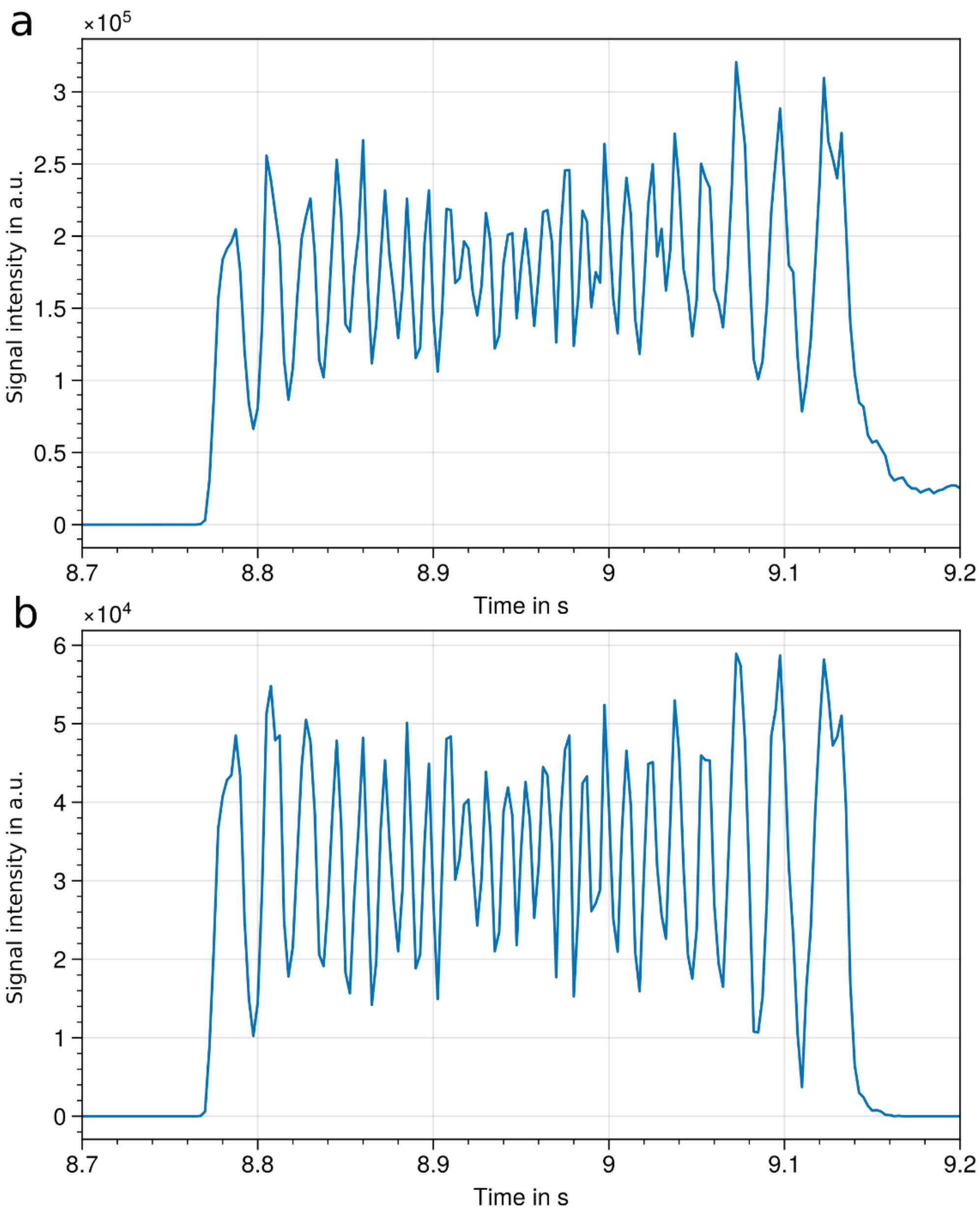

Figure S5: LAP-MALDI MS analysis of HHL using a 1536-well plate layout. a) total ion chromatogram, and b) extracted ion chromatogram at 60 samples/s.

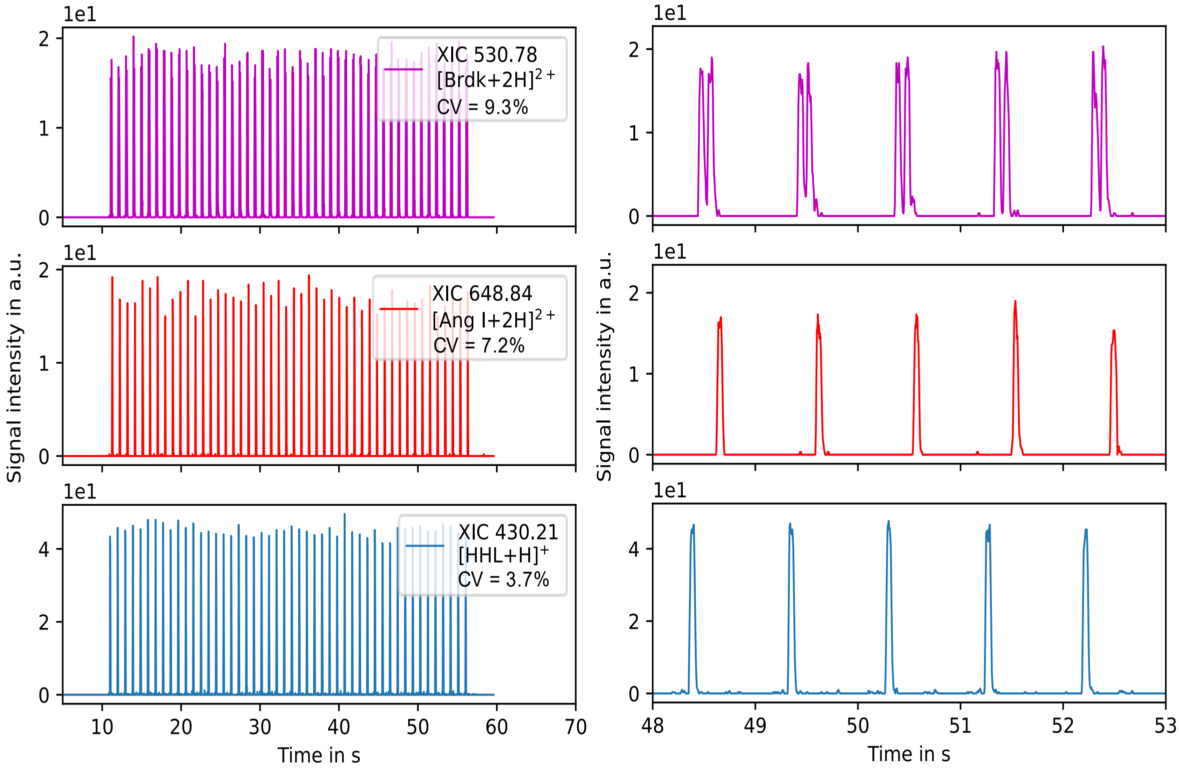

Figure S6: Variability of peptide analysis in TDC mode. 48 consecutive LAP-MALDI analyses of 2x2 sample spots of Brdk and different standards at start and end at 50 mm/s stage speed. Enlargement on right side.

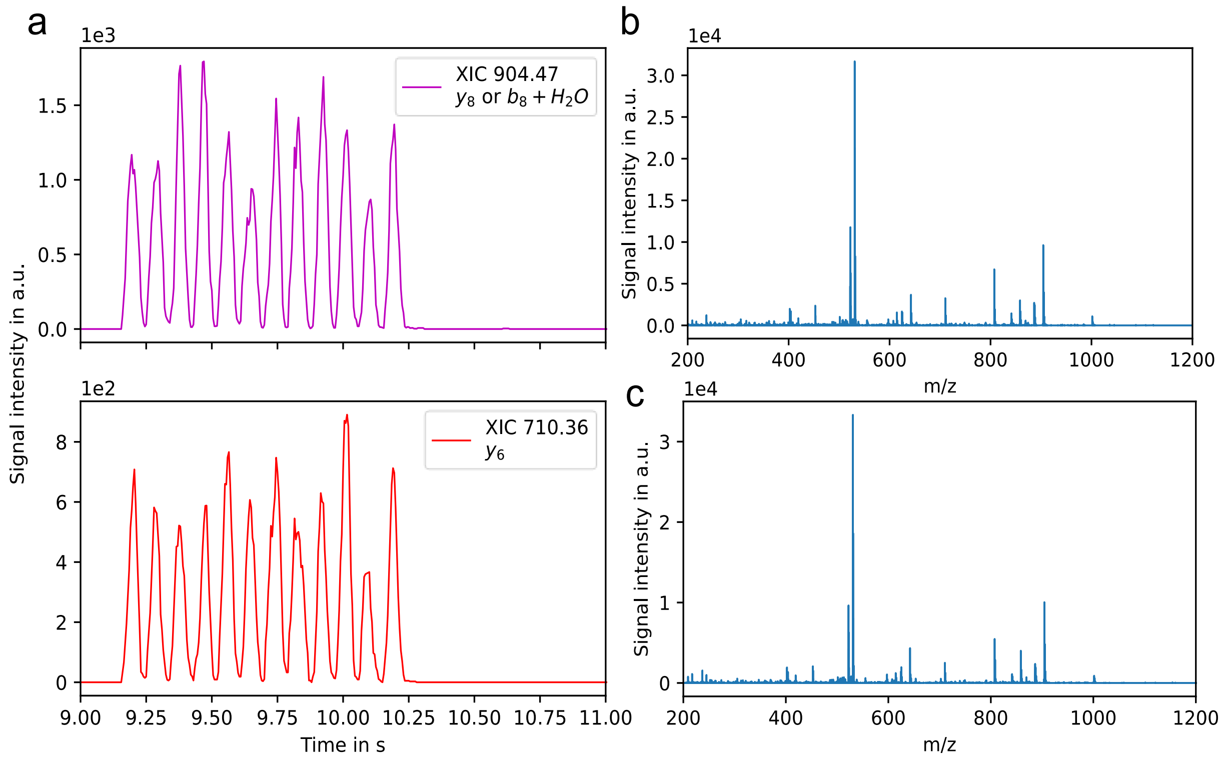

Figure S7: Tandem mass spectrometry at 50 mm/s. 12 samples of Brdk with quadrupole filtering around m/z 530.78 and trap collisional voltage at 25 V. a) Extracted ion chromatogram of two fragment ions; b) Mass spectrum of first sample; c) Mass spectrum of last sample.
